# Regional network covariance patterns of white matter integrity related to cardiorespiratory fitness in healthy aging

**DOI:** 10.3389/fnagi.2025.1542458

**Published:** 2025-06-26

**Authors:** Samantha G. Smith, Pradyumna K. Bharadwaj, David A. Raichlen, Matthew D. Grilli, Jessica R. Andrews-Hanna, Georg A. Hishaw, Matthew J. Huentelman, Theodore P. Trouard, Gene E. Alexander

**Affiliations:** ^1^Department of Psychology, University of Arizona, Tucson, AZ, United States; ^2^Evelyn F. McKnight Brain Institute, University of Arizona, Tucson, AZ, United States; ^3^Human and Evolutionary Biology Section, Department of Biological Sciences, University of Southern California, Los Angeles, CA, United States; ^4^Department of Anthropology, University of Southern California, Los Angeles, CA, United States; ^5^Department of Neurology, University of Arizona, Tucson, AZ, United States; ^6^Cognitive Science Program, University of Arizona, Tucson, AZ, United States; ^7^Neurogenomics Division, The Translational Genomics Research Institute (TGen), Phoenix, AZ, United States; ^8^Arizona Alzheimer’s Consortium, Phoenix, AZ, United States; ^9^Department of Biomedical Engineering, University of Arizona, Tucson, AZ, United States; ^10^Department of Psychiatry, University of Arizona, Tucson, AZ, United States; ^11^Neuroscience Graduate Interdisciplinary Program, University of Arizona, Tucson, AZ, United States; ^12^Physiological Sciences Graduate Interdisciplinary Program, University of Arizona, Tucson, AZ, United States

**Keywords:** diffusion-weighted imaging, VO_2_max, brain aging, scaled subprofile model, multivariate analyses

## Abstract

Cardiorespiratory fitness (CRF), measured by VO_2_max, is an indicator of vascular functioning that can influence the integrity of brain microstructural white matter tracts in aging. How CRF is related to regional patterns of white matter bundles for magnetic resonance imaging (MRI) diffusion metrics (axial diffusivity, AD; radial diffusivity, RD; mean diffusivity, MD; fractional anisotropy, FA) has been less studied. We used a multivariate analysis method, the Scaled Subprofile Model (SSM), to identify network patterns of MRI tract-specific white matter integrity (WMI) for AD, RD, MD, and FA related to VO_2_max and to evaluate their relation to demographic, vascular health, and dementia risk factors in 167 cognitively unimpaired older adults, ages 50 to 88. We identified four CRF-related regional patterns of WMI characterized by enhanced integrity in commissural pathways that connect areas within anterior brain regions (prefrontal body of the corpus callosum), connect subcortical regions to one another (fornix), and include selected association tracts (arcuate fasciculus, superior longitudinal fasciculus). Greater white matter lesion load, in addition to age, was associated with reduced expression of all four CRF-WMI patterns, while high vascular risk level was further associated with reduced expression of the RD, MD, and FA patterns. The regional patterns of RD and FA were most strongly associated with CRF. The results suggest that in healthy older adults, enhanced CRF is differentially associated with regional patterns of WMI, which are related to age and further impacted by macrostructural white matter lesion load and vascular risk. These findings support the use of the multivariate SSM in identifying regional patterns of white matter tracts that may provide markers of brain aging and cerebrovascular health.

## 1 Introduction

Advanced age is associated with greater incidence of cardiovascular disease (Rodgers et al., 2019). Maintaining vascular health in old age may help to reduce the effects of brain aging. Cardiovascular health conditions have been shown to negatively impact structural brain volumes and quality of life, as well as increase the risk for dementia (Kivipelto et al., 2001; Viswanathan et al., 2009). Directly assessing cardiorespiratory fitness (CRF) by obtaining VO_2_max (maximal oxygen consumption) through cardiopulmonary exercise testing can provide an important indicator of vascular health. Both genetic factors and modifiable lifestyle behaviors are significant contributors to the composition and trainability of CRF, as well as its role in healthy aging (Bouchard et al., 2015; Zadro et al., 2017; Sarzynski et al., 2022).

Aging in the absence of cognitive impairment or dementia (i.e., healthy aging) has been associated with differences in brain structure (Alexander et al., 2006; Raz and Rodrigue, 2006; Farokhian et al., 2017), including the integrity of white matter tracts. Magnetic resonance imaging (MRI) metrics of microstructural white matter integrity (WMI) derived from diffusion-weighted imaging have included measures of axial diffusivity (AD), radial diffusivity (RD), mean diffusivity (MD), and fractional anisotropy (FA). These have been suggested to reflect aspects of axonal loss and demyelination in the context of healthy aging (Lu et al., 2011; Salat, 2011; Soares et al., 2013). Reductions in RD and MD have consistently been shown to reflect enhanced WMI while increases in FA indicate better tract integrity. AD’s association with aging has been more variable, with age-related increases and decreases being reported (Bennett et al., 2010; Burzynska et al., 2010).

In healthy older adults, greater VO_2_max has been associated with enhanced microstructural integrity in tracts connecting regions in the frontal and parietal lobes (Colcombe et al., 2006; Gordon et al., 2008; Voss et al., 2013). Greater CRF levels have been related to enhanced FA and decreased RD (Johnson et al., 2012; Zhu et al., 2015), even in the absence of significant effects of physical activity levels on WMI metrics (Chen et al., 2020; Strömmer et al., 2020; d’Arbeloff et al., 2020). CRF and engagement in physical activity have also been shown to have distinct associations with aspects of structural and functional brain measures, including gray matter volume and thickness and functional connectivity (Voss et al., 2016; Raichlen et al., 2020; Olivo et al., 2021), such that CRF has been associated with enhanced brain structure and function separate from physical activity. The associations have also involved white matter tracts connected to anterior brain regions, including the genu of the corpus callosum, superior longitudinal fasciculus, inferior longitudinal fasciculus, and uncinate fasciculus (Johnson et al., 2012; Hayes et al., 2015; Oberlin et al., 2016; Ding et al., 2018). These studies have typically relied on univariate analytic methods that may limit the ability to fully characterize the regionally distributed associations of CRF with WMI in the context of healthy aging. Further research, using multivariate statistical methods that may be more sensitive in identifying patterns of tract-specific regional brain differences (Alexander and Moeller, 1994; Gazes et al., 2016; Geeraert et al., 2019, 2020), may help to better elucidate the relation of CRF to WMI in bundles preferentially vulnerable to aging. Such multivariate network analyses provide a way to test regional WMI tract associations with CRF, without the need to control for multiple comparisons while having the opportunity to statistically adjust for related health and demographic characteristics. Given previous evidence of preferential alterations for selected white matter tracts with age, evaluating multivariate regional patterns of tract-specific metrics of integrity may help to better characterize the potential influence of CRF on regional WMI in the healthy aging population (Bennett and Madden, 2014; de Groot et al., 2015; Bender et al., 2016).

Clinical vascular health risk factors (e.g., hypertension, hyperlipidemia, smoking, and diabetes) increase the risk of cerebrovascular disease (CVD) and can adversely impact WMI (Fuhrmann et al., 2019). Poor vascular health has also been shown to increase risk for developing Alzheimer’s disease (Meng et al., 2014; O’Brien and Markus, 2014). The common genetic risk factor for late-onset Alzheimer’s disease, the apolipoprotein E (*APOE*) ε4 allele, has specifically been associated with increased risk of coronary heart disease and CVD, as well as detrimental effects on white matter (Kaprio et al., 1991; Fullerton et al., 2000; Raichlen and Alexander, 2014; Wang et al., 2015). Multiple cardiovascular risk factors often co-exist in older adults (Genest Jr and Cohn, 1995). There is evidence that the contributions of these risk factors can be additive, such that the risk of cognitive impairment increases with each additional risk factor (Luchsinger et al., 2005). Moreover, having a greater number of cardiovascular risk factors has been associated with greater brain atrophy and poorer white matter health (Cox et al., 2019). Among older adults, the presence of multiple vascular risk factors has been associated with greater reductions in cerebral blood flow compared to those with one or no vascular risk factors and has been further associated with poorer cognitive performance (Bangen et al., 2014). White matter hyperintensity (WMH) volumes on MRI, that reflect chronic small vessel disease, increase with both age and with vascular health risk factors and may be an indicator of disrupted WMI (Gunning-Dixon and Raz, 2000; Habes et al., 2016).

The present study used a multivariate network covariance analysis method, the Scaled Subprofile Model (SSM; Moeller et al., 1987; Alexander and Moeller, 1994) to identify regional patterns of WMI related to CRF, as measured by VO_2_max, to evaluate which white matter bundles and which integrity metrics (i.e., FA, MD, RD, AD) may be most sensitive to differences in CRF in healthy aging. The multivariate SSM has been used in many structural neuroimaging studies to identify patterns of regional gray matter differences associated with age and multiple health factors (Alexander et al., 2006, 2008, 2012, 2020; Bergfield et al., 2010; Kern et al., 2017; Song et al., 2023; Van Etten et al., 2024). Differences in WMI using the SSM have been evaluated in relation to aging and cognitive function (Gazes et al., 2016), but this analytic method has yet to be directly applied to CRF with regional measures of WMI. We sought to apply multivariate SSM to directly investigate the relationship between CRF and regional tracts of WMI in the context of healthy aging. This study also sought to examine how (1) demographics (i.e., age and sex), (2) vascular health and dementia risk, including clinical vascular risk factors and *APOE* ε4 carrier status, and (3) WMH lesion load were each associated with the identified regional SSM network patterns of CRF-related WMI. We hypothesized that greater integrity in white matter bundles connecting anterior and posterior brain regions, such as the genu of the corpus callosum and superior longitudinal fasciculus, would be related to elevated CRF. Further, we hypothesized that patterns of CRF-related WMI would be negatively impacted by poorer vascular health.

## 2 Methods

### 2.1 Participants

Healthy adults (*N* = 167; Age = 69.0 ± 10.4 years) ages 50–88, underwent cardiopulmonary exercise testing and brain MRI scans. The sample was cognitively unimpaired, as indicated by the Mini Mental Status Exam (MMSE = 28.98 ± 1.26; Folstein et al., 1975), and included 78 females (46.7%) and predominantly (93.4%) non-Hispanic white participants. To exclude significant neurological, medical, and psychiatric disorders, participants underwent an extensive medical screen, and a physical and neurological examination performed by a neurologist (GAH) who specializes in aging and dementia. Participants were excluded if they had a MMSE score less than 26 or a Hamilton Depression Rating Scale (HAM-D; Hamilton, 1960) score greater than 9. The present participants comprise a subsample of a larger cohort of 210 older adults, including only those who were able to complete a treadmill test of CRF and diffusion-weighted MRI scans. Fourteen participants were excluded based on inability to complete VO_2_max testing, 16 additional participants were excluded due to not undergoing neuroimaging, and 13 participants were excluded based on poor data quality for the MRI scans (described in Section 2.5.1). Sixty participants (35.9%) were determined to have high cardiovascular risk based on endorsement of two or more available risk factors: history of cardiac arrest, hypertension, high cholesterol, diabetes, and historical or current smoker status (Bangen et al., 2014; Boutzoukas et al., 2022; see Table 1). All procedures were approved by the Institutional Review Board at the University of Arizona and all participants provided informed written consent.

**Table 1:** Participant characteristics.

*N*	167
Age [years; M (SD)]	68.99 (10.41)
Education [years; M (SD)]	15.76 (2.58)
Sex (F/M)	78/89
MMSE [M (SD)]	28.98 (1.26)
VO_2_max [ml/kg/min; M (SD)]	24.43 (5.40)
Cardiac arrest (Y/N)	5/161^†^
Hypertension (Y/N)	55/112
Hyperlipidemia (Y/N)	73/94
Diabetes (Y/N)	8/159
Smoking history (Y/N)	66/101
Vascular risk level (H/L)	60/107
*APOE ε*4 Status (Y/N)	49/118

Means (standard deviations) for the sample. F/M, Female/Male; Y/N, Yes/No; MMSE, Mini Mental Status Exam; *APOE*, apolipoprotein E ε4; H/L, High/Low, High = two or more vascular risk factors, Low = less than two vascular risk factors. ^†^One participant with missing data for cardiac arrest history (*n* = 166).

### 2.2 Exercise testing

Participants completed a treadmill graded exercise test at the Pulmonary Function Laboratory at the University of Arizona Medical Center (Tucson, AZ). Each treadmill session began at a low intensity with a speed of 1 mph and an incline grade of 0%. Speed and grade were gradually increased based on the modified Naughton treadmill protocol during exercise (Berry et al., 1996; Strzelczyk et al., 2001). Expiratory gasses were measured using a metabolic cart and standard techniques of open-circuit spirometry and oxygen uptake was obtained using indirect calorimetry. Maximal oxygen consumption (VO_2_max [ml/kg/min]) to assess CRF was considered achieved when two of the following three criteria were satisfied: (1) plateau in VO_2_ with an increase in workload; (2) respiratory exchange ratio of 1:1 (VCO_2_: VO_2_) or higher; and (3) heart rate within 10 beats of the age-predicted maximum. Termination criteria were consistent with the guidelines of the American College of Sports Medicine (Pescatello et al., 2014).

### 2.3 Genetic testing

*APOE* genotyping was performed at the Translational Genomics Research Institute (TGen, Phoenix, AZ, United States) from venous blood samples and was determined using restriction-fragment-length polymorphisms (RFLP), which has been described in previous studies (Van Etten et al., 2021; Song et al., 2023). Briefly, high molecular weight DNA were extracted, assayed, and then amplified using AmpliTaq Gold Fast PCR Master Mix (Applied Biosystems: Thermo Fisher Scientific, Waltham, MA). Samples were assessed for characteristic banding patterns of the six common *APOE* genotypes according to previously published methods (Addya et al., 1997). In the present cohort, there were 49 *APOE* ε4 carriers (29.3%) and 118 non-carriers (70.7%).

### 2.4 Image acquisition

T1-weighted Spoiled Gradient Echo (SPGR) MRI scans (slice thickness = 1.0 mm, TR = 5.3 ms, TE = 2.0 ms, TI = 500 ms, flip angle = 15°, matrix = 256×256, FOV = 25.6 cm) and T2 Fluid-Attenuated Inversion Recovery (FLAIR) scans (slice thickness = 2.6 mm, TR = 11,000 ms, TE = 120 ms, TI = 2,250 ms, flip angle = 90°, matrix = 256×256, FOV = 25.0 cm) were acquired on a 3 T GE Signa scanner (HD Signa Excite, General Electric, Milwaukee, WI). Diffusion-weighted images (DWI) in 51 directions with 8 B0 images (b = 1,000 s/mm2, slice thickness = 2.6 mm, TR = 12,500 ms, TE = 70 ms, flip angle = 90°, matrix = 128 × 128, FOV = 25 cm, and 58 slices) were also acquired during the same MRI session.

### 2.5 Image processing

#### 2.5.1 Diffusion tensor imaging

Pre-processing of diffusion MRI data involved eddy current correction (Smith et al., 2004) and EPI distortion correction using Brainsuite’s INVERSION method (Bhushan et al., 2015). These images were processed using TRACULA (Yendiki et al., 2011; Maffei et al., 2021) for automated global probabilistic tractography of 42 white matter bundles using a diffusion model capable of modeling crossing fibers (Behrens et al., 2007) and extraction of standard diffusivity measures (AD, RD, MD, and FA). Briefly, this involved using the specific structural brain regions of interest created by the FreeSurfer processing stream (Fischl et al., 2002) in conjunction with a manually labeled training dataset of WM pathway priors to estimate the posterior probability distribution for the 42 WM tracts for each participant. To delineate the seed regions, TRACULA uses the specified WM pathway endpoints from the training set and transforms them into each individual’s native space (Yendiki et al., 2011; Maffei et al., 2021). Streamlines from the seed regions are generated by TRACULA with a Bayesian framework that models the entire pathway globally, using splines simultaneously fitted to the diffusion orientation data from voxels along the length of the pathway. Voxel-wise diffusion orientation is produced using the multi-compartment ball-and-stick model (Behrens et al., 2007), which is capable of modeling crossing fibers. Curvature constraints for pathways are implicitly derived from the control points mapped from the training data to the native space, along with neighborhood information along the pathway (Yendiki et al., 2011; Maffei et al., 2021). This modeling procedure leverages regional neighborhood information along the length of the tract, produced by FreeSurfer’s automated cortical parcellation and subcortical segmentation of each participant’s corresponding T1-weighted MRI scan (Fischl et al., 2002; Yendiki et al., 2011; Maffei et al., 2021). Quality control procedures for outputs of TRACULA involved visual inspection of tracts flagged for low volume (< 1 SD below the mean). Such tracts were re-initialized to re-sample from their posterior distribution. Participants were excluded if: (1) Visual inspection of re-initialized tracts continued to include anatomically mis-specified or partially reconstructed streamlines, (2) during visual inspection, any tract contained just one streamline or many incorrect streamlines, (3) more than one of the four diffusion metrics were extreme values (beyond 3 IQR) for a tract, or (4) any of the four diffusion metrics were extreme values (beyond 3 IQR) on more than one tract. Based on these parameters, 13 participants were excluded for poor DWI data quality. Estimates of total intracranial volume (TIV) were obtained in native brain space from each of the T1 images using Statistical Parametric Mapping (SPM12; Wellcome Trust Center for Neuroimaging, London, United Kingdom) by calculating the sum of the total gray matter, white matter, and cerebrospinal fluid segments (Alexander et al., 2012).

#### 2.5.2 WMH volume

The volume of WMH lesions were computed using T1 and T2-FLAIR scans and the lesion segmentation toolbox (LST; Schmidt et al., 2012) with SPM12. WMH probability maps were generated with the multispectral lesion growth algorithm by LST in a subset of the sample at a range of values for the optimization parameter kappa and spatially compared with reference WMH maps to determine the optimal threshold (0.35) for the present cohort of cognitively unimpaired older adults (Franchetti et al., 2020; Van Etten et al., 2021). Voxel volumes in the WMH maps were summed to compute the total WMH volume in milliliters (ml) and the global values were log transformed for subsequent analyses (Franchetti et al., 2020; Van Etten et al., 2021).

### 2.6 Statistical analyses

#### 2.6.1 Network covariance patterns

Regional SSM network analysis (Moeller et al., 1987; Alexander and Moeller, 1994; Habeck et al., 2008; Alexander et al., 2012) was performed using MATLAB (Math Works, Natick, Massachusetts, United States). First, diffusivity metrics for each white matter bundle underwent natural log transformation and mean values across regions and participants were subtracted at each tract. Next, a principal component analysis (PCA) was performed, producing a set of regional covariance pattern components and corresponding network subject scores, which reflected the degree to which each participant expressed the identified regional pattern. The Bayesian Information Criterion (BIC; Schwarz, 1978) was used as a model selection method to identify the best set of SSM components for each diffusion metric. This method was chosen as it accounts for sample size differences in selecting the best model, providing a conservative selection of components for identifying the covariance pattern that may provide for greater generalizability and reproducibility of the network pattern across samples (Dziak et al., 2020). A bootstrap re-sampling procedure (Efron and Tibshirani, 1994) with 10,000 iterations was applied in the SSM analysis (Gazes et al., 2016; Habeck et al., 2008; Alexander et al., 2012) to provide reliability estimates for the regional white matter integrity values for the observed pattern weights related to tract-specific diffusion metrics and VO_2_max. The linearly combined SSM pattern weights with bootstrap resampling provide information on the meaningful contribution of each regional white matter bundle to the SSM patterns for each of the four WMI metrics. Influence of TIV was subsequently assessed for each WMI SSM pattern using multiple regression to test the association of CRF-related WMI pattern expression with VO_2_max after adjusting for TIV. Standardized regression coefficients were used to aid interpretation across the four WMI metrics. Network analyses were also followed by univariate regression for the individual tracts in relation to VO_2_max for the four diffusion metrics to assess how each tract identified as significant in the SSM analyses contributed to the CRF-related network patterns, with false discovery rate (FDR) correction for multiple comparisons (Benjamini and Hochberg, 1995).

#### 2.6.2 Regressions with demographic and health factors

Block-wise multiple linear regression analyses in SPSS (v22, Chicago, IL, USA) were used to test how demographic and vascular health and dementia risk factors were associated with expression of each CRF-related WMI pattern. While adjusting for TIV in block one, age and sex were entered into block two to evaluate their association with CRF-related WMI pattern expression. Vascular risk level (0 or 1 vs. 2 or more clinical vascular risk factors) and *APOE* ε4 status (e4 non-carrier vs. carrier) were then added in block three to evaluate their associations with expression of the SSM patterns. Global WMH volume was subsequently added as the final covariate in block 4 to evaluate the impact of macrostructural white matter lesion load on the models.

Follow-up sensitivity analyses were conducted by adding individual vascular risk factors (i.e., history of cardiac arrest, hypertension, high cholesterol, diabetes, and historical or current smoker status) to the models in place of vascular risk level to (1) assess for differential contributions of individual risk factors to expression of the CRF-WMI patterns and (2) evaluate the potential unique additive effect of vascular risk factors on the network patterns. Follow-up analyses were also performed with use of hypertensive medication status as a separate covariate to further assess potential hypertension effects.

## 3 Results

### 3.1 Network covariance patterns

**Figure 1:**
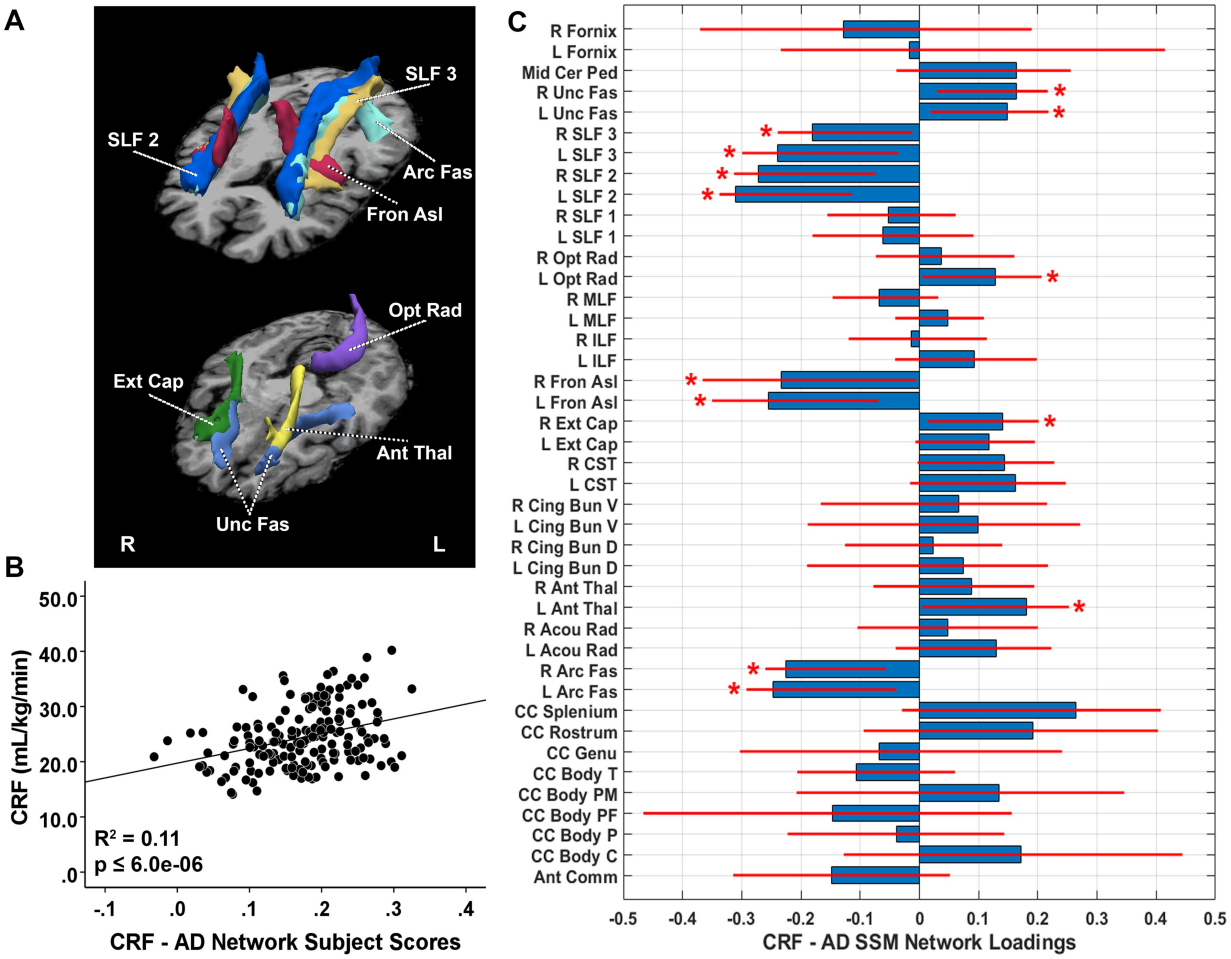
**(A)** White matter tracts identified as significant loadings in the cardiorespiratory fitness (CRF)-related axial diffusivity (AD) network pattern. **(B)** CRF-AD network subject scores and CRF. The subject scores of the CRF-AD network pattern derived from the first six SSM components. The scatterplots show that greater CRF was associated with higher expression of the network pattern. Adjusted *R^2^* and *p* values are displayed. **(C)** CRF-related tract-specific AD loadings for the SSM network pattern of AD. Blue bars indicate point estimates for the loadings and red lines indicate the 95% confidence intervals. Asterisks reflect significant ROIs contributing to the covariance pattern. SSM, Scaled Subprofile Model; R, right; L, left; Mid Cer Ped, middle cerebellar peduncle; Unc Fas, uncinate fasciculus; SLF, superior longitudinal fasciculus; Opt Rad, optic radiation; MLF, middle longitudinal fasciculus; ILF, inferior longitudinal fasciculus; Fron Asl, frontal aslant; Ext Cap, external capsule; CST, corticospinal tract; Cing Bun V, cingulum bundle ventral; Cing Bun D, cingulum bundle dorsal; Ant Thal, anterior thalamic radiation; Acou Rad, acoustic radiation; Arc Fas, arcuate fasciculus; CC, corpus callosum; Body T, temporal body; Body PM, premotor body; Body PF, prefrontal body; Body P, body parietal; Body C, body central; Ant Comm, anterior commissure.

The SSM analyses with BIC model selection criteria identified the linear combination of components that best predicted CRF (i.e., VO_2_max [ml/kg/min]) for each diffusion metric. The CRF-related AD pattern included the first six components and accounted for 11.2% of the variance in CRF (*β* = 0.342, *p* = 6.00E-7) with higher expression of the network pattern associated with greater CRF. Bootstrap resampling of the linearly combined regional pattern was characterized by reductions of AD in the bilateral arcuate fasciculus, bilateral frontal aslant, and bilateral superior longitudinal fasciculus (SLF) 2 and 3 tracts, with relative increases in the left anterior thalamic radiation, right external capsule, left optic radiation, and bilateral uncinate fasciculus tracts (Figure 1). After adjustment for TIV, pattern expression remained significantly associated with CRF (*β* = 0.313, adjusted *R*^2^ change = 0.094, *p* = 1.20E-5). Follow-up univariate analyses revealed that AD in bilateral arcuate fasciculus, bilateral frontal aslant, bilateral SLF 2, and bilateral SLF 3 were negatively associated with VO_2_max, indicating greater WMI in these tracts with enhanced CRF (Table 2). Tracts identified as relative increases in the SSM pattern all showed significant decreases univariately: left anterior thalamic radiation, right external capsule, left optic radiation, and bilateral uncinate fasciculus, suggesting that these tracts reflect relatively less enhanced integrity with increasing CRF (Table 2).

**Table 2:** Univariate associations of CRF with white matter tracts within SSM network patterns.

CRF-related pattern	White matter tract	*β*	*p*-FDR	95% confidence interval
AD	L Arc Fas	−0.334	2.90E-5	(−53.0, −20.9)
	R Arc Fas	−0.346	1.60E-5	(−50.8, −20.9)
	L Fron Asl	−0.345	1.60E-5	(−49.2, −20.2)
	R Fron Asl	−0.337	2.60E-5	(−47.9, −19.1)
	L SLF 2	−0.328	3.70E-5	(−42.7, −16.5)
	R SLF 2	−0.297	1.76E-4	(−39.3, −13.3)
	L SLF 3	−0.315	7.30E-5	(−48.5, −17.8)
	R SLF 3	−0.350	1.20E-5	(−53.1, −22.2)
	L Ant Thal	−0.252	0.001	(−49.4, −12.8)
	R Ext Cap	−0.207	0.008	(−50.8, −7.99)
	L Opt Rad	−0.165	0.033	(−41.8, −1.75)
	L Unc Fas	−0.193	0.013	(−51.6, −6.30)
	R Unc Fas	−0.237	0.003	(−58.9, −13.3)
RD	CC Body PF	−0.463	2.96E-10	(−30.4, −16.6)
	L Arc Fas	−0.292	2.00E-4	(−36.8, −12.2)
	R Arc Fas	−0.307	1.00E-4	(−41.0, −14.6)
	L Acou Rad	−0.198	0.011	(−29.6, −4.02)
	R Acou Rad	−0.219	0.005	(−34.2, −6.42)
	L SLF 1	−0.315	7.30E-5	(−33.8, −12.4)
	L SLF 3	−0.296	1.79E-4	(−35.2, −11.8)
	R SLF 3	−0.384	2.00E-6	(−43.0, −19.8)
	L CST	−0.036	0.641	(−15.9, 9.81)
	R CST	−0.056	0.473	(−16.6, 7.76)
MD	CC Body PF	−0.446	1.51E-9	(−41.7, −22.0)
	CC Genu	−0.368	5.00E-6	(−39.7, −17.5)
	L Fron Asl	−0.371	5.00E-6	(−44.0, −19.6)
	R Fron Asl	−0.369	5.00E-6	(−43.2, −19.1)
	L Fornix	−0.292	2.00E-4	(−13.7, −4.51)
	L Acou Rad	−0.231	0.004	(−41.3, −8.84)
	R Acou Rad	−0.251	0.001	(−42.8, −11.0)
	L Opt Rad	−0.275	4.67E-4	(−43.6, −13.1)
	R Opt Rad	−0.313	7.80E-5	(−46.3, −16.8)
	Mid Cer Ped	−0.250	0.001	(−43.4, −11.0)
	L CST	−0.122	0.118	(−32.8, 3.71)
	R CST	−0.119	0.126	(−33.9, 4.21)
FA	CC Body PF	0.346	1.51E-9	(13.1, 31.8)
	R Fornix	0.351	1.20E-5	(9.92, 23.7)
	R Arc Fas	0.041	0.599	(−8.62, 14.9)
	R Acou Rad	−0.012	0.876	(−15.0, 12.8)
	L CST	−0.110	0.155	(−28.1, 4.52)
	R CST	−0.046	0.554	(−20.5, 11.0)
	R MLF	0.042	0.593	(−10.0, 17.5)

White matter tracts identified in the CRF-WMI SSM network patterns. β represents the standardized coefficient. False discovery rate (FDR) corrected p values are displayed. Key: Acou Rad, acoustic radiation; AD, axial diffusivity; Ant Thal, anterior thalamic radiation; Arc Fas, arcuate fasciculus; Body PF, prefrontal body; CC, corpus callosum; CRF, cardiorespiratory fitness; CST, corticospinal tract; Ext Cap, external capsule; FA, fractional anisotropy; Fron Asl, frontal aslant; L, left; MD, mean diffusivity; Mid Cer Ped, middle cerebellar peduncle; MLF, middle longitudinal fasciculus; Opt Rad, optic radiation; RD, radial diffusivity; R, right; SLF, superior longitudinal fasciculus; SSM, Scaled Subprofile Model; Unc Fas, uncinate fasciculus.

**Figure 2:**
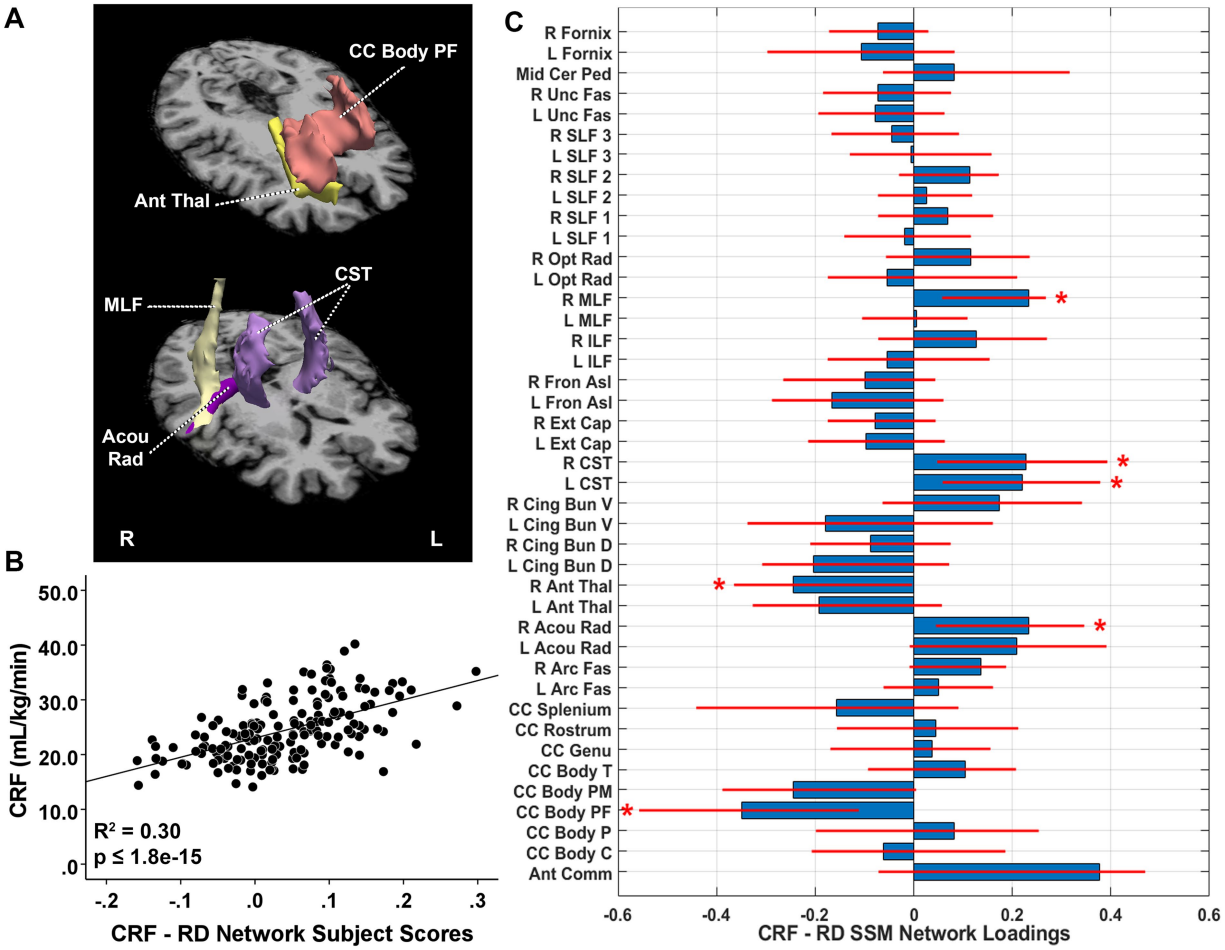
**(A)** White matter tracts identified as significant loadings in the cardiorespiratory fitness (CRF)-related radial diffusivity (RD) network pattern. **(B)** CRF-RD network subject scores and CRF. The subject scores of the CRF-RD network pattern were derived from the first four SSM components. The scatterplots show that greater CRF was associated with higher expression of the network pattern. Adjusted *R^2^* and *p* values are displayed. **(C)** CRF-related tract-specific RD loadings for the SSM network pattern of RD. Blue bars indicate point estimates for the loadings and red lines indicate the 95% confidence intervals. Asterisks reflect significant ROIs contributing to the covariance pattern. SSM, Scaled Subprofile Model; R, right; L, left; Mid Cer Ped, middle cerebellar peduncle; Unc Fas, uncinate fasciculus; SLF, superior longitudinal fasciculus; Opt Rad, optic radiation; MLF, middle longitudinal fasciculus; ILF, inferior longitudinal fasciculus; Fron Asl, frontal aslant; Ext Cap, external capsule; CST, corticospinal tract; Cing Bun V, cingulum bundle ventral; Cing Bun D, cingulum bundle dorsal; Ant Thal, anterior thalamic radiation; Acou Rad, acoustic radiation; Arc Fas, arcuate fasciculus; CC, corpus callosum; Body T, temporal body; Body PM, premotor body; Body PF, prefrontal body; Body P, body parietal; Body C, body central; Ant Comm, anterior commissure.

The CRF-related RD pattern included the first four components and accounted for 30.2% of the variance in VO_2_max (*β* = 0.553, *p* = 8.88E-15) with higher expression of the network pattern related to greater CRF. This pattern was characterized by reductions in RD (lower RD indicates better tract integrity) in the prefrontal body of the corpus callosum with relative increases in the bilateral arcuate fasciculus, bilateral acoustic radiation, bilateral corticospinal tract (CST), left SLF 1, and bilateral SLF 3 bundles (Figure 2). After adjusting for TIV, pattern expression was significantly associated with CRF (*β* = 0.511, adjusted *R*^2^ change = 0.253, *p* = 9.21E-14). Univariately, the prefrontal body of the corpus callosum was negatively associated with CRF. Tracts showing relative increases in the SSM pattern were also negatively associated with CRF, including the bilateral arcuate fasciculus, bilateral acoustic radiation, left SLF 1, and bilateral SLF 3. Bilateral CST was not significantly related to CRF (Table 2). These results suggest that the relative reductions in the prefrontal body of the corpus callosum observed in the SSM RD pattern reflects greater integrity in relation to increasing CRF, whereas the tracts identified as relative increases in the SSM pattern reflect relatively less enhanced WMI with greater CRF.

**Figure 3:**
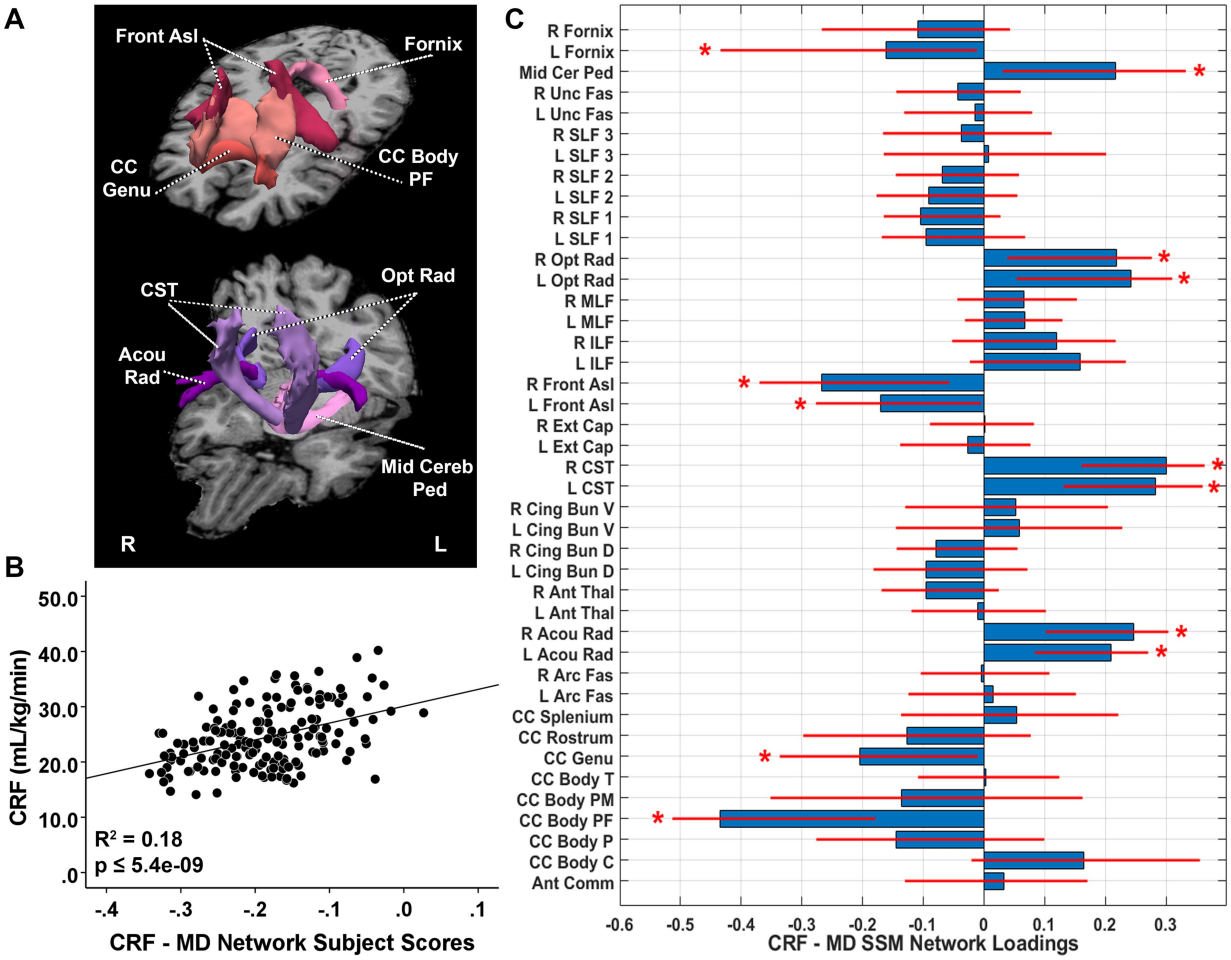
**(A)** White matter tracts identified as significant loadings in the cardiorespiratory fitness (CRF)-related mean diffusivity (MD) network pattern. **(B)** CRF-MD network subject scores and CRF. The subject scores of the CRF-MD network pattern were derived from the first six SSM components. The scatterplots show that greater CRF was associated with higher expression of the network pattern. Adjusted *R^2^* and *p* values are displayed. **(C)** CRF-related tract-specific MD loadings for the SSM network pattern of RD. Blue bars indicate point estimates for the loadings and red lines indicate the 95% confidence intervals. Asterisks reflect significant ROIs contributing to the covariance pattern. SSM, Scaled Subprofile Model; R, right; L, left; Mid Cer Ped, middle cerebellar peduncle; Unc Fas, uncinate fasciculus; SLF, superior longitudinal fasciculus; Opt Rad, optic radiation; MLF, middle longitudinal fasciculus; ILF, inferior longitudinal fasciculus; Fron Asl, frontal aslant; Ext Cap, external capsule; CST, corticospinal tract; Cing Bun V, cingulum bundle ventral; Cing Bun D, cingulum bundle dorsal; Ant Thal, anterior thalamic radiation; Acou Rad, acoustic radiation; Arc Fas, arcuate fasciculus; CC, corpus callosum; Body T, temporal body; Body PM, premotor body; Body PF, prefrontal body; Body P, body parietal; Body C, body central; Ant Comm, anterior commissure.

The CRF-related MD pattern included the first six components and accounted for 18.2% of the variance in VO_2_max (*β* = 0.432, *p* = 5.45E-9) with higher expression of the network pattern related to greater CRF. The pattern was characterized by reductions in MD (lower values indicate better tract integrity) in the prefrontal body of the corpus callosum, the genu of the corpus callosum, bilateral frontal aslant, and left fornix bundles with relative increases in the bilateral acoustic radiation, bilateral CST, bilateral optic radiation, and middle cerebellar peduncle (Figure 3). After TIV adjustment, pattern expression was significantly associated with CRF (*β* = 0.412, adjusted *R*^2^ change = 0.166, *p* = 4.05E-9). Follow-up univariate analyses revealed that MD in the prefrontal body of the corpus callosum, genu of the corpus callosum, bilateral frontal aslant, and left fornix were significantly negatively associated with VO_2_max. MD in tracts identified as relative increases in the SSM pattern showed significant univariate decreases with CRF, including the bilateral acoustic radiation, bilateral optic radiation, and middle cerebellar peduncle. MD values in bilateral CST were not significantly associated with VO_2_max (Table 2). White matter bundles identified as relative decreases in the SSM MD pattern reflect regions with greater integrity with increasing CRF, while those identified as relative increases reflect tracts with relatively less enhanced integrity with greater CRF.

**Figure 4:**
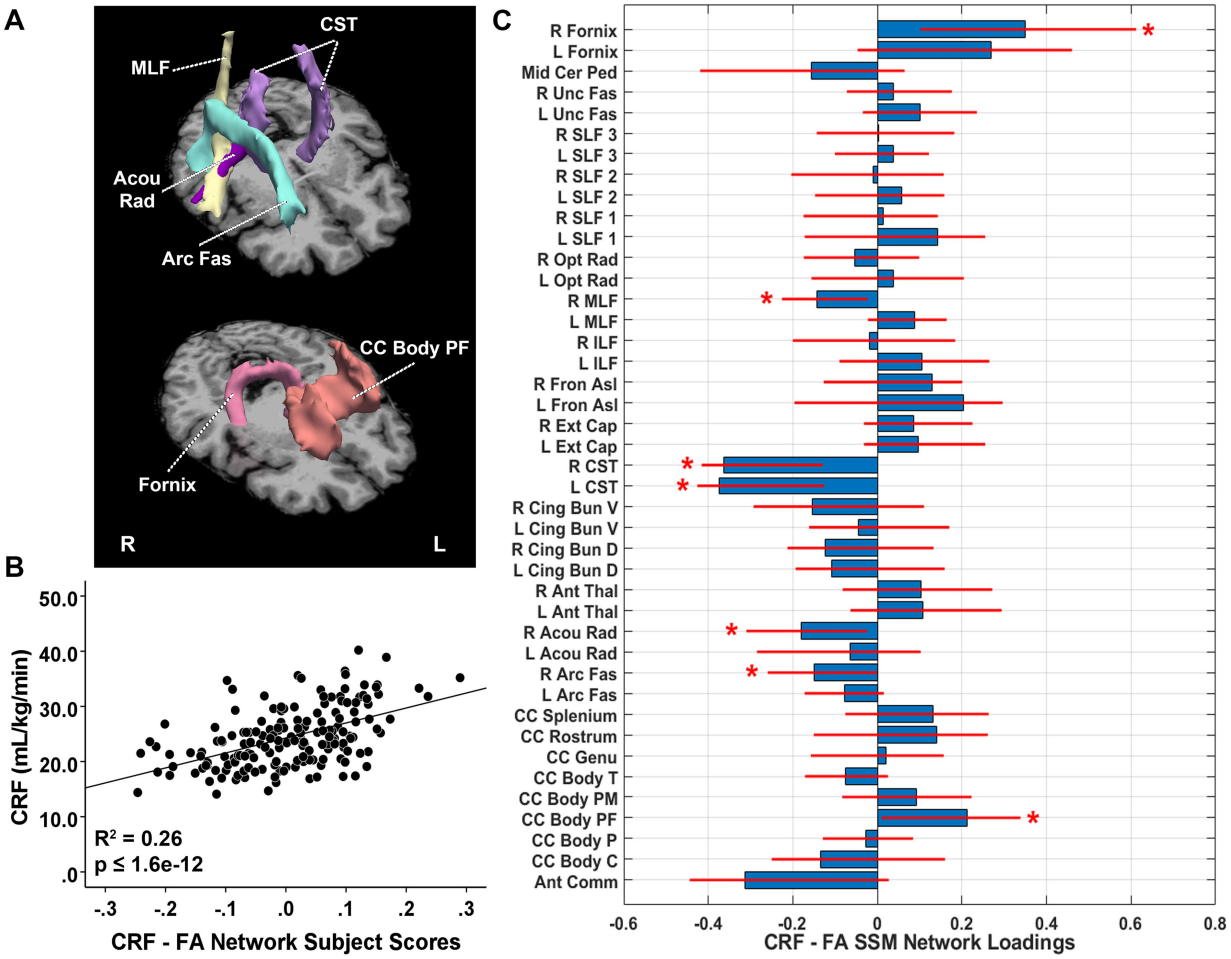
**(A)** White matter tracts identified as significant loadings in the cardiorespiratory fitness (CRF)-related fractional anisotropy (FA) network pattern. **(B)** CRF-FA network subject scores and CRF. The subject scores of the CRF-FA network pattern were derived from the first eight SSM components. The scatterplots show that greater CRF was associated with higher expression of the network pattern. Adjusted *R^2^* and *p* values are displayed. **(C)** CRF-related tract-specific FA loadings for the SSM network pattern of RD. Blue bars indicate point estimates for the loadings and red lines indicate the 95% confidence intervals. Asterisks reflect significant ROIs contributing to the covariance pattern. SSM, Scaled Subprofile Model; R, right; L, left; Mid Cer Ped, middle cerebellar peduncle; Unc Fas, uncinate fasciculus; SLF, superior longitudinal fasciculus; Opt Rad, optic radiation; MLF, middle longitudinal fasciculus; ILF, inferior longitudinal fasciculus; Fron Asl, frontal aslant; Ext Cap, external capsule; CST, corticospinal tract; Cing Bun V, cingulum bundle ventral; Cing Bun D, cingulum bundle dorsal; Ant Thal, anterior thalamic radiation; Acou Rad, acoustic radiation; Arc Fas, arcuate fasciculus; CC, corpus callosum; Body T, temporal body; Body PM, premotor body; Body PF, prefrontal body; Body P, body parietal; Body C, body central; Ant Comm, anterior commissure.

The CRF-related FA pattern included the first eight components and accounted for 25.7% of the variance in CRF (*β* = 0.512, *p* = 1.60E-12) with higher expression of the network pattern related to greater fitness. The pattern was characterized by relative reductions in FA (higher FA indicates better tract integrity) in the right arcuate fasciculus, right acoustic radiation, bilateral CST, and right middle longitudinal fasciculus (MLF) with increases in the prefrontal body of the corpus callosum and right fornix bundles (Figure 4). After adjustment for TIV, pattern expression remained significantly associated with CRF (*β* = 0.478, adjusted *R*^2^ change = 0.223, *p* = 3.94E-12). Follow-up univariate analyses revealed that FA in the prefrontal body of the corpus callosum and right fornix were significantly positively associated with VO_2_max (Table 2). No FA values in tracts identified in the pattern as relative decreases were significantly associated with VO_2_max univariately, including right arcuate fasciculus, right acoustic radiation, bilateral CST, and right MLF (Table 2). These results suggest that the observed relative increases in the SSM FA pattern in the prefrontal body of the corpus callosum and right fornix reflect greater WMI with increasing CRF, whereas those areas identified as relative FA decreases in the pattern reflect relatively less enhanced tract integrity with increasing CRF.

Overall, the network covariance patterns consistently identified several white matter bundles that demonstrated greater tract integrity with greater VO_2_max values across multiple WMI diffusion metrics, including the prefrontal body of the corpus callosum, genu of the corpus callosum, bilateral fornix, bilateral arcuate fasciculus, and bilateral frontal aslant. The SSM patterns also identified covarying white matter bundles that are relatively less associated with greater CRF, indicating relatively less enhanced integrity with increasing levels of VO_2_max, including the bilateral CST, bilateral acoustic radiation, and bilateral uncinate fasciculus.

### 3.2 Demographics and vascular health risk factors

Expression of each CRF-related WMI network pattern was then tested with multiple linear regression for associations with TIV (block 1), age and sex (block 2), vascular risk level and *APOE* ε4 status (block 3), and global WMH volume (block 4). Full results for each pattern are shown in Table 3 (AD pattern results), 4 (RD pattern results), 5 (MD pattern results), and 6 (FA pattern results). Only CRF-related RD pattern expression was significantly associated with TIV in the first models (AD: *β* = 0.093, *p* = 0.231; RD: *β* = 0.163, *p* = 0.035; MD: *β* = 0.066, *p* = 0.396; FA: *β* = 0.117, *p* = 0.131). After adding age and sex to the models, age was significantly inversely related for all SSM patterns (AD: *β* = −0.390, *p* = 5.02E-7; RD: *β* = −0.547, *p* = 1.00E-13; MD: *β* = −0.470, *p* = 8.38E-10; FA: *β* = −0.628, *p* = 2.93E-18). Neither TIV (AD: *β* = −0.084, *p* = 0.379; RD: *β* = 0.052, *p* = 0.546; MD: *β* = −0.078, *p* = 0.397; FA: *β* = −0.073, *p* = 0.369) nor sex (AD: *β* = 0.182, *p* = 0.055; RD: *β* = 0.035, *p* = 0.679; MD: *β* = 0.108, *p* = 0.235; FA: *β* = 0.142, *p* = 0.080) were significant predictors for any patterns in model 2 (Tables 3–6).

**Table 3:** Summary of multiple regression analyses for variables predicting the CRF-AD network pattern.

Variable	Model 1				Model 2				Model 3				Model 4			
	*β*	B	SE	*p*	*β*	B	SE	*p*	*β*	B	SE	*p*	*β*	B	SE	*p*
TIV	0.093	0.066	0.055	0.231	−0.084	−0.059	0.067	0.379	−0.095	−0.067	0.068	0.326	−0.031	−0.022	0.069	0.752
Age					**−0.390**	**−0.003**	**0.000**	**5.02E-7**	**−0.385**	**−0.003**	**0.000**	**8.78E-7**	**−0.221**	**−0.001**	**0.001**	**0.020**
Sex					0.182	0.025	0.013	0.055	**0.193**	**0.027**	**0.013**	**0.045**	**0.199**	**0.027**	**0.013**	**0.035**
Vascular risk level									−0.058	−0.008	0.011	0.434	−0.064	−0.009	0.010	0.377
*APOE*									−0.019	−0.003	0.011	0.797	−0.007	−0.001	0.011	0.919
WMH													**−0.260**	**−0.041**	**0.015**	**0.005**
Adjusted *R*^2^	0.003				0.139				0.133				0.169			
F for *R*^2^	1.44			0.231	**9.97**			**5.00E-6**	**6.08**			**3.50E-5**	**6.61**			**3.00E-6**

β represents the standardized coefficient and B the unstandardized coefficient. SE indicates t and standard error of natural log-transformed B. Sex, vascular risk level, and *APOE* ε4 status were dummy coded 1 for males, high vascular risk level, and APOE ε4 carriers, respectively. Bolded variables are significant (*p* < 0.05). Key: APOE, apolipoprotein E; TIV, total intracranial volume; WMH, white matter hyperintensity volume.

**Table 4:** Summary of multiple regression analyses for variables predicting the CRF-RD network pattern.

Variable	Model 1				Model 2				Model 3				Model 4			
	*β*	B	SE	*p*	*β*	B	SE	*p*	*β*	B	SE	*p*	*β*	B	SE	*p*
TIV	**0.163**	**0.142**	**0.067**	**0.035**	0.052	0.045	0.075	0.546	0.035	0.031	0.075	0.686	0.105	0.092	0.075	0.221
Age					**−0.547**	**−0.004**	**0.001**	**1.00E-13**	**−0.533**	**−0.004**	**0.001**	**3.39E-13**	**−0.353**	**−0.003**	**0.001**	**3.30E-5**
Sex					0.035	0.006	0.014	0.679	0.060	0.010	0.015	0.484	0.066	0.011	0.014	0.423
Vascular risk level									**−0.132**	**−0.023**	**0.012**	**0.048**	**−0.139**	**−0.025**	**0.011**	**0.032**
*APOE*									0.018	0.003	0.012	0.786	0.030	0.006	0.012	0.632
WMH													**−0.285**	**−0.055**	**0.016**	**5.99E-4**
Adjusted *R*^2^	0.021				0.300				0.308				0.353			
F for *R*^2^	**4.51**			**0.035**	**24.7**			**3.28E-13**	**15.8**			**1.25E-12**	**16.1**			**1.87E-14**

β represents the standardized coefficient and B the unstandardized coefficient. SE indicates t and standard error of natural log-transformed B. Sex, vascular risk level, and *APOE* ε4 status were dummy coded 1 for males, high vascular risk level, and *APOE* ε4 carriers, respectively. Bolded variables are significant (*p* < 0.05). Key: *APOE*, apolipoprotein E; TIV, total intracranial volume; WMH, white matter hyperintensity volume.

**Table 5:** Summary of multiple regression analyses for variables predicting the CRF-MD network pattern.

Variable	Model 1				Model 2				Model 3				Model 4			
	*β*	B	SE	*p*	*β*	B	SE	*p*	*β*	B	SE	*p*	*β*	B	SE	*p*
TIV	0.066	0.052	0.061	0.396	−0.078	−0.061	0.072	0.397	−0.100	−0.079	0.072	0.279	−0.053	−0.042	0.074	0.572
Age					**−0.470**	**−0.003**	**0.001**	**8.38E-10**	**−0.456**	**−0.003**	**0.001**	**2.27E-9**	**−0.336**	**−0.002**	**0.001**	**2.91E-4**
Sex					0.108	0.017	0.014	0.235	0.135	0.021	0.014	0.143	0.139	0.021	0.014	0.126
Vascular risk level									−0.140	−0.022	0.011	0.051	**−0.144**	**−0.023**	**0.011**	**0.041**
*APOE*									−0.012	−0.002	0.012	0.863	−0.004	−0.001	0.012	0.958
WMH													**−0.191**	**−0.033**	**0.016**	**0.034**
Adjusted *R*^2^	−0.002				0.196				0.206				0.223			
F for *R*^2^	0.723			0.396	**14.5**			**2.09E-8**	**9.60**			**4.95E-8**	**8.94**			**2.06E-8**

β represents the standardized coefficient and B the unstandardized coefficient. SE indicates t and standard error of natural log-transformed B. Sex, vascular risk level, and *APOE* ε4 status were dummy coded 1 for males, high vascular risk level, and APOE ε4 carriers, respectively. Bolded variables are significant (*p* < 0.05). Key: APOE, apolipoprotein E; TIV, total intracranial volume; WMH, white matter hyperintensity volume.

**Table 6:** Summary of multiple regression analyses for variables predicting the CRF-FA network pattern.

Variable	Model 1				Model 2				Model 3				Model 4			
	*β*	B	SE	*p*	*β*	B	SE	*p*	*β*	B	SE	*p*	*β*	B	SE	*p*
TIV	0.117	0.122	0.080	0.131	−0.073	−0.076	0.085	0.369	−0.098	−0.102	0.084	0.229	−0.057	−0.059	0.086	0.489
Age					**−0.628**	**−0.006**	**0.001**	**2.93E-18**	**−0.612**	**−0.006**	**0.001**	**8.82E-18**	**−0.508**	**−0.005**	**0.001**	**1.81E-9**
Sex					0.142	0.029	0.016	0.080	**0.172**	**0.035**	**0.016**	**0.033**	**0.176**	**0.036**	**0.016**	**0.028**
Vascular risk level									**−0.161**	**−0.034**	**0.013**	**0.010**	**−0.165**	**−0.035**	**0.013**	**0.008**
*APOE*									−0.007	−0.001	0.014	0.915	0.001	0.000	0.014	0.990
WMH													**−0.165**	**−0.038**	**0.018**	**0.036**
Adjusted *R*^2^	0.008				0.371				0.389				0.402			
F for *R*^2^	2.31			0.131	**33.6**			**5.88E-17**	**22.1**			**7.81E17**	**19.6**			**4.66E-17**

β represents the standardized coefficient and B the unstandardized coefficient. SE indicates t and standard error of natural log-transformed B. Sex, vascular risk level, and *APOE* ε4 status were dummy coded 1 for males, high vascular risk level, and APOE ε4 carriers, respectively. Bolded variables are significant (*p* < 0.05). Key: APOE, apolipoprotein E; TIV, total intracranial volume; WMH, white matter hyperintensity volume.

After subsequently adding vascular risk and *APOE* ε4 status to the models, we found that vascular risk level was inversely associated with expression of the RD and FA patterns (AD: *β* = −0.058, *p* = 0.434; RD: *β* = −0.132, *p* = 0.048; MD: *β* = −0.140, *p* = 0.051; FA: *β* = −0.161, *p* = 0.010) while *APOE* ε4 status did not significantly contribute predictive value for any patterns (AD: *β* = −0.019, *p* = 0.797; RD: *β* = 0.018, *p* = 0.786; MD: *β* = −0.012, *p* = 0.863; FA: *β* = −0.007, *p* = 0.915). In these models, age remained significant across all patterns (AD: *β* = −0.385, *p* = 8.78E-7; RD: *β* = −0.533, *p* = 3.39E-13; MD: *β* = −0.456, *p* = 2.27E-9; FA: *β* = −0.612, *p* = 8.82E-18), sex emerged as a significant predictor for AD and FA patterns with greater pattern expression associated with male sex (AD: *β* = 0.193, *p* = 0.045; RD: *β* = 0.060, *p* = 0.484; MD: *β* = 0.135, *p* = 0.143; FA: *β* = 0.172, *p* = 0.033), and TIV was not a significant predictor for all regional diffusion patterns (AD: *β* = −0.095, *p* = 0.326; RD: *β* = 0.035, *p* = 0.686; MD: *β* = −0.100, *p* = 0.279; FA: *β* = −0.098, *p* = 0.229).

Finally, adding global WMH volume to the models to assess the association of macrostructural WMI with expression of the CRF-related WMI patterns revealed that WMH volume was significantly negatively associated with SSM participant scores across all diffusion patterns (AD: *β* = −0.260, *p* = 0.005; RD: *β* = −0.285, *p* = 5.99E-4; MD: *β* = −0.191, *p* = 0.034; FA: *β* = −0.165, *p* = 0.036). In these final models, TIV remained non-significant across all patterns (AD: *β* = −0.031, *p* = 0.752; RD: *β* = 0.105, *p* = 0.221; MD: *β* = −0.053, *p* = 0.572; FA: *β* = −0.057, *p* = 0.489), age remained a significant predictor across all patterns (AD: *β* = −0.221, *p* = 0.020; RD: *β* = −0.353, *p* = 3.30E-5; MD: *β* = −0.336, *p* = 2.91E-4; FA: *β* = −0.508, *p* = 1.81E-9), sex remained a significant predictor in AD and FA pattern models (AD: *β* = 0.199, *p* = 0.035; RD: *β* = 0.066, *p* = 0.423; MD: *β* = 0.139, *p* = 0.126; FA: *β* = 0.176, *p* = 0.028), vascular risk level emerged as a significant predictor for expression of the MD pattern and remained a significant predictor for the RD and FA patterns (AD: *β* = −064, *p* = 0.377; RD: *β* = −0.139, *p* = 0.032; MD: *β* = −0.144, *p* = 0.041; FA: *β* = −0.165, *p* = 0.008), and *APOE* ε4 status remained non-significant across all patterns (AD: *β* = −0.007, *p* = 0.919; RD: *β* = 0.030, *p* = 0.632; MD: *β* = −0.004, *p* = 0.958; FA: *β* = 0.001, *p* = 0.990). In follow-up sensitivity analyses, no individual vascular risk factors (*p*’s > 0.05) or use of hypertensive medication (*p* > 0.05) was significantly associated with CRF-WMI pattern expression. Significant results were unchanged after additionally controlling for the time interval between exercise testing and MRI acquisition (25.9 ± 3.14 days), which was not associated with expression of any CRF-WMI pattern (*p*’s > 0.05).

## 4 Discussion

The present study used a multivariate network covariance approach to identify regional patterns of tract-specific WMI associated with CRF in a healthy aging cohort. Across the identified regional patterns of diffusion WMI metrics, including for AD, RD, MD, and FA, greater VO_2_max was associated with enhanced integrity in white matter bundles primarily connecting anterior portions of the brain, as well as in wider association areas. Multivariate patterns of MD and FA additionally identified white matter bundles connecting subcortical brain regions related to greater CRF. These results extend previous studies, which have identified both frontal (Gordon et al., 2008; Voss et al., 2013) and subcortical associations of CRF with WMI in tracts vulnerable to brain aging (Davis et al., 2009; Burgmans et al., 2010; Meier et al., 2012; Tian et al., 2014).

Specifically, relative reductions of AD were observed in white matter tracts connecting frontal regions to temporal and parietal regions, as well as connecting inferior frontal gyrus to superior frontal gyrus, indicating better integrity with increased CRF. The pattern also identified relative increases, which reflected comparatively less decreases relative to greater CRF based on univariate follow-up analyses, in tracts connecting the thalamus to frontal and occipital cortices, tracts connecting basal ganglia structures, and those connecting temporal and frontal regions. While previous studies have not always found CRF associations with AD (Johnson et al., 2012; Voss et al., 2013), the results are consistent with other studies that have demonstrated a relationship between AD and CRF in SLF 1 (Ding et al., 2018). AD, a measurement of diffusivity that extends parallel to WM tracts, is generally thought to reflect axonal integrity (Concha et al., 2006). Disruptions to AD may additionally be influenced by macrostructural effects, such as the formation of WMH, due to disrupted diffusion of water molecules (Bennett et al., 2010; Salat, 2011). This was shown in the follow-up regression analyses in the present study, where expression of the CRF-AD pattern was significantly negatively associated with global WMH volume while accounting for age, sex, and cardiovascular and dementia risk factors (i.e., vascular risk level, *APOE* status). Although a decrease in AD can also be seen following acute axonal injury (Budde et al., 2009), the present study sample was screened to exclude individuals with significant neurologic conditions that may be more associated with these types of acute infarcts. The reduced AD relative to increased CRF for selected tracts connecting frontal, temporal, and parietal brain regions in the present results may be more likely to reflect enhanced tract integrity for AD, which is also consistent with other reports in healthy aging (Sullivan et al., 2008; Zahr et al., 2008; Kumar et al., 2013). Further research is needed to help clarify the directional relation of AD with enhanced integrity in healthy older adults. These findings additionally indicate that multivariate statistical methods, like the SSM, may be able to detect subtle regional differences in tract-specific WMI related to CRF that univariate methods may not.

The CRF-RD pattern revealed relative reductions in the prefrontal body of the corpus callosum (connecting rostral middle frontal regions between hemispheres) with relative increases, which again appeared as comparatively less decreases, in tracts connecting frontal to temporal regions, thalamus to temporal regions, spinal tract to frontal areas, and anterior to posterior regions. These results are consistent with previous work that found lower RD associated with greater CRF levels in the corpus callosum and pre-motor areas (Johnson et al., 2012; Tarumi et al., 2022). RD is commonly thought to reflect myelin integrity through measurement of diffusion perpendicular to WM tracts (Song et al., 2023). Thus, the CRF-RD pattern may indicate WM bundles with enhanced or preserved myelination related to greater CRF in addition to bundles with preferentially less reductions in diffusivity. Additionally, expression of the pattern was significantly associated with age, vascular risk level, and global WMH volume, such that greater pattern expression was related to younger age, lower vascular risk, and lower overall WMH lesion load. Compared to the CRF-AD pattern, the CRF-RD pattern was more strongly associated with cardiovascular health in this healthy older adult cohort.

The CRF-MD pattern was characterized by reductions in the prefrontal body and genu of the corpus callosum (connecting rostral middle and mid-anterior frontal regions between hemispheres) and tracts connecting frontal regions to one another as well as connecting hippocampal and midbrain structures with relative increases in tracts connecting the thalamus to temporal and occipital areas, spinal tract to frontal regions, and cerebellar hemispheres to the contralateral cortex. MD represents a combination of AD and RD, reflecting the average rate (Madden et al., 2009; Bennett and Madden, 2014), magnitude (Salat, 2011), and motility of water diffusion, independent of the directionality (Sullivan et al., 2008). The CRF-MD pattern displayed significant overlap with the AD and RD patterns but also uniquely detected CRF associations in the fornix connecting subcortical regions. Similarly to the CRF-RD pattern, greater expression of the CRF-MD was significantly associated with younger age, lower cardiovascular risk levels, and lower global WMH volume.

Finally, unlike AD, RD, and MD, higher values of FA indicate better directional tract integrity. The CRF-FA pattern was characterized by increases in the prefrontal body of the corpus callosum and bundles connecting hippocampal and midbrain structures. The pattern also identified relative reductions (i.e., tracts with comparatively less increases in FA) in the tract connecting the thalamus, parietal regions, and frontal regions with temporal areas as well as connecting spinal tract to frontal regions. Although FA is sensitive to directional white matter microstructural differences, it lacks specificity for the type of white matter alterations that may be present (Alexander et al., 2011). These findings are consistent with previous studies showing that CRF levels have been related to enhanced FA in tracts connecting anterior brain regions (Johnson et al., 2012; Zhu et al., 2015). The pattern also suggests a potential hemispheric difference in FA for associations with CRF in the right hemisphere. Greater expression of this pattern was significantly associated with younger age, male sex, lower vascular health risk, and reduced macrostructural white matter lesion load.

While the patterns differed for some identified tracts, several were shared across diffusion metric patterns, including the prefrontal body of the corpus callosum, fornix, and superior longitudinal fasciculus, suggesting these bundles may be especially sensitive to CRF-related differences across several MRI diffusion measures of WMI. Results additionally suggest that RD and FA may be most sensitive to differences in the integrity of white matter related to risk factors for cardiovascular health, as these patterns were most strongly associated with both VO_2_max and vascular risk level in this cohort of healthy older adults. This is consistent with previous studies that have found significant associations with CRF in both RD and FA in the absence of AD and MD relationships (Johnson et al., 2012). Together, these findings support network regional covariance patterns of brain white matter tracts sensitive to CRF differences in healthy aging. Moreover, these patterns may represent useful neuroimaging biomarkers of cardiovascular health in older age.

Previous literature has been mixed on the extent to which CRF impacts white matter in healthy aging, as well as which bundles are sensitive to differences in cardiovascular health. Multivariate statistical approaches have been useful for detecting local, tract-specific associations of aging and CRF, while reducing the need for multiple comparisons and accounting for shared variance between closely related white matter tracts (De Santis et al., 2014a; Geeraert et al., 2019, 2020). The present results indicate that greater CRF was mainly associated with preferential preservation of WMI in the prefrontal body and genu of the corpus callosum, arcuate fasciculus, superior longitudinal fasciculus, frontal aslant tract, and fornix, suggesting that these bundles are sensitive to CRF differences in a cognitively unimpaired, generally healthy aging sample. Follow-up regression analyses revealed that all CRF-related WMI patterns were significantly associated with age, with increasing age related to reduced expression of the patterns, and each reflecting greater WMI with increasing CRF. Additionally, reduced expression of the RD, MD, and FA patterns was related to vascular risk level, such that those with at least two clinical vascular risk factors displayed less preservation of the CRF-related WMI above and beyond effects of age, sex, and TIV. Notably, greater expression of the AD and FA patterns in males was observed after adjusting for other covariates. Future research is warranted for evaluation of potential sex differences in WMI related to CRF. Although previous work has shown detrimental effects of *APOE* ε4 on WMI (Heise et al., 2024), none of the present CRF-WMI patterns were significantly associated with *APOE* ε4 status in our generally healthy cognitively unimpaired cohort. These results provide support for multiple clinical vascular risk factors associated with added risk for brain aging. It has been hypothesized that CRF may attenuate age-related cognitive decline via increased delivery and upregulation of neurotrophins and other supporting factors in brain regions particularly vulnerable to demyelination in old age (Stimpson et al., 2018). Other potential mechanisms include increased cerebral perfusion, synaptogenesis, and angiogenesis (Maass et al., 2015; Tsai et al., 2016; Stimpson et al., 2018). CVD and associated risk factors may interfere with these processes. Thus, high CRF may help attenuate age-related effects on myelin and subsequently associated cognitive functions. Follow-up research studies are needed to determine the impact of CRF on white matter and associated aspects of cognition. Longitudinal data would be especially important to better understand the relationship between CRF and WMI over time and the extent to which this relationship can be modified by different lifestyle factors (e.g., diet, exercise).

Global WMH volume was a significant predictor of all four SSM CRF-related patterns while we controlled for age, sex, vascular health, and *APOE* ε4 carrier status. These findings suggest that chronic small vessel disease may further disrupt WMI sensitive to CRF in older age, distinct from the accumulation of common cardiovascular health risk factors. Although the exact mechanisms remain unclear, it has been suggested that WMH can disrupt local and distal WMI by alterations in water mobility in the interstitial space (Wardlaw et al., 2013), increased blood–brain barrier permeability (Farrall and Wardlaw, 2009), and/or Wallerian degeneration (Pierpaoli et al., 2001).

The present study has several limitations. First, the sample was largely homogenous with primarily non-Hispanic white, highly educated participants with relatively low cardiovascular health burden compared to the general population of older adults. Given evidence of racial/ethnic and socioeconomic disparities in WMI outcomes (Weiss et al., 2024), further research with more diverse samples in dimensions of race/ethnicity, education, socioeconomic status, and cardiovascular health is needed to further evaluate the generalizability of our findings. Additionally, the present results rely on evaluating the associations of CRF with brain metrics from one time point, which does not allow for the assessment of causality. Future research would benefit from evaluating longitudinal changes in CRF and WMI in older adults to better understand the dynamics of these relationships throughout both the healthy and pathological aging process. It would be important for future studies to evaluate whether and how lifestyle behavioral interventions can help to modify these dynamics, as well as the extent to which they are associated with cognitive and clinical outcomes. Finally, further research should utilize additional neuroimaging measures with increased specificity for alterations in microstructural white matter characteristics (e.g., axonal density) to enhance precision of WMI measurement and to further clarify specific mechanisms in the associations between CRF and WMI in aging (de Santis et al., 2014b).

## 5 Conclusion

The present study used a multivariate covariance approach to identify regional network patterns of tract-specific WMI for brain diffusion measures of AD, RD, MD, and FA related to CRF in cognitively unimpaired older adults. The resulting patterns were characterized by enhanced WMI in relation to greater CRF across all four diffusion metrics, involving white matter bundles mainly connecting anterior brain regions as well as wider association tracts. Greater expression of these patterns was also strongly and consistently associated with younger age and less macrostructural white matter lesion load; and higher expression of several network patterns (i.e., RD, MD, and FA) were related to lower levels of vascular risk, suggesting differential associations of WMI diffusion metrics with cardiovascular health. Together, these results highlight unique contributions from the presence of multiple vascular risk factors and age on brain WMI in healthy older adults. Finally, our analyses support the use of multivariate network analyses, like SSM, with MRI diffusion metrics of localized white matter tracts as potential neuroimaging markers of lifestyle influences on brain aging that can help to assess relationships between CRF, WMI, age, and cardiovascular health in the context of healthy aging.

## Data Availability

The raw data supporting the conclusions of this article will be made available by the authors, without undue reservation.
